# Functional Integration of Grafted Neural Stem Cell-Derived Dopaminergic Neurons Monitored by Optogenetics in an *In Vitro* Parkinson Model

**DOI:** 10.1371/journal.pone.0017560

**Published:** 2011-03-04

**Authors:** Jan Tønnesen, Clare L. Parish, Andreas T. Sørensen, Angelica Andersson, Cecilia Lundberg, Karl Deisseroth, Ernest Arenas, Olle Lindvall, Merab Kokaia

**Affiliations:** 1 Experimental Epilepsy Group, Wallenberg Neuroscience Center, Lund University Hospital, Lund, Sweden; 2 Laboratory of Molecular Neurobiology, Department of Medical Biochemistry and Biophysics, Center for Developmental Biology and Regenerative Medicine, Karolinska Institute, Stockholm, Sweden; 3 CNS Gene Therapy Unit, Wallenberg Neuroscience Center, Lund University Hospital, Lund, Sweden; 4 Department of Bioengineering, Stanford University, Stanford, California, United States of America; 5 Laboratory of Neurogenesis and Cell Therapy, Wallenberg Neuroscience Center, Lund University Hospital, Lund, Sweden; National Institute on Aging Intramural Research Program, United States of America

## Abstract

Intrastriatal grafts of stem cell-derived dopamine (DA) neurons induce behavioral recovery in animal models of Parkinson's disease (PD), but how they functionally integrate in host neural circuitries is poorly understood. Here, *Wnt5a*-overexpressing neural stem cells derived from embryonic ventral mesencephalon of tyrosine hydroxylase-GFP transgenic mice were expanded as neurospheres and transplanted into organotypic cultures of wild type mouse striatum. Differentiated GFP-labeled DA neurons in the grafts exhibited mature neuronal properties, including spontaneous firing of action potentials, presence of post-synaptic currents, and functional expression of DA D_2_ autoreceptors. These properties resembled those recorded from identical cells in acute slices of intrastriatal grafts in the 6-hydroxy-DA-induced mouse PD model and from DA neurons in intact substantia nigra. Optogenetic activation or inhibition of grafted cells and host neurons using channelrhodopsin-2 (ChR2) and halorhodopsin (NpHR), respectively, revealed complex, bi-directional synaptic interactions between grafted cells and host neurons and extensive synaptic connectivity within the graft. Our data demonstrate for the first time using optogenetics that ectopically grafted stem cell-derived DA neurons become functionally integrated in the DA-denervated striatum. Further optogenetic dissection of the synaptic wiring between grafted and host neurons will be crucial to clarify the cellular and synaptic mechanisms underlying behavioral recovery as well as adverse effects following stem cell-based DA cell replacement strategies in PD.

## Introduction

Generation of dopamine (DA) neurons for transplantation from stem cells of various sources has gained substantial interest for restorative therapy in Parkinson's disease (PD) [1], [2], [3]. The reversal of PD symptoms following intrastriatal implantation of such cells in animal models raises questions about the cellular and synaptic mechanisms responsible for functional recovery, particularly the level of synaptic integration of the stem cell-derived DA neurons into host neural circuitries. With the exception of mouse embryonic stem (ES) cell-derived DA neurons [4], [5], the extent of DA fiber outgrowth has been limited and the afferent inputs to the grafted DA cells have not been determined. Despite restricted fiber outgrowth, ventral midbrain neurospheres overexpressing *Wnt5a*, a member of the Wnt family that is known to promote DA neuron differentiation [6], induced a robust behavioral response in the 6-hydroxy-DA-induced mouse PD model [7]. However, it has been difficult to analyze the functional synaptic integration of the grafted neurons into host neural circuitries due to lack of experimental approaches for discriminating between interspersed host and graft cells, and to specifically target these two cell groups for electrophysiological analysis of their interconnectivity. Therefore, it is not known to what extent stem cell-derived DA neurons establish efferent synaptic connections with host neurons or receive functional synaptic inputs from other grafted cells or host neurons.

Here, we have investigated, using patch-clamp recordings and optogenetic tools, the functional properties and synaptic integration of DA neurons, generated from neural stem/progenitor cells in mouse ventral mesencephalic neurospheres (VMNs), when grafted into striatum of organotypic mouse hemisphere slice cultures. These hemisphere cultures can be considered as *in vitro* model of PD, since midbrain dopaminergic inputs from substantia nigra to striatum are severed by slicing and degenerate, while intrastriatal connectivity and cortical synaptic inputs are mostly preserved [8], [9].

## Methods

### Animals

Mice of different strains (Balb/c, TH-GFP/C57bl6 heterozygotes, C57bl6 and CD-1 immunodefficient) were housed under 12 h light/12 h dark cycle with *ad libitum* access to food and water. All experiments were approved by Lund/Malmö (permit M85/06) and Stockholm (permits N150/05 and N154/06) ethical committees.

### Ventral midbrain neurospheres

Neural stem cell neurospheres were prepared as previously described [7]. In brief, ventral midbrains were isolated from embryonic day 10.5 (E10.5) mice, generated by crossing TH-GFP x C57bl6 parents, yielding a population of TH-GFP-expressing, presumed DA neurons upon differentiation [10], [11]. Ventral midbrains were mechanically triturated and cultured as neurospheres in an atmosphere of 5% CO_2_ and 3% O_2_ at 37°C in the presence of FGF2, FGF8 (each 20 ng/ml, R&D Systems) and Sonic hedgehog (Shh, 500 ng/ml, R&D Systems) in DMEM/F12 (Gibco) supplemented with Albumax (3 mg/ml, Gibco), glucose (6 mg/ml, Sigma), glutamine (1 mM, Gibco) and 1% N2 supplement (Gibco) [7]. The resulting neural stem cell neurospheres were expanded by passaging using collagenase/dispase for dissociations (700 µg/ml, Roche) once or twice before *in vitro* grafting on striatal organotypic slice cultures.

### *Wnt5a* and pCAIP2 transfections

Plasmid transfections were performed as described in detail earlier [7]. In brief, three days after passage 1 or 2, neurospheres were transfected with *Wnt5a* (VMN-*Wnt5a*) or the empty control pCAIP2 plasmid (VMN) using Lipofectamine 2000 (Invitrogen). Sodium butyrate (1 mM; Chemicon) was added 4 h after transfections to enhance promoter activity [12]. The following day, neurospheres were transferred to fresh medium for an additional 24 h before co-culturing.

To confirm increased *Wnt5a* expression, a Q-PCR expression assay was applied on 10.000–50.000 transfected cells the day after transfections. Cells were washed in DMEM/F12 medium, transferred to RLT lysis buffer (Quiagen) and mercaptoethanol 0.1 µl/ml. Q-PCR was performed with a commercially available *Wnt5a* expression assay (TaqMan Mm00437347_m1; Applied Biosystems). Expression was normalized to the housekeeping gene Glyceraldehyde 3-phosphate dehydrogenase (GADPH) and expressed relative to non-transfected cells [13].

### Organotypic striatal cultures and co-culture

Organotypic cultures were prepared as 250 µm thick coronal hemisphere sections of postnatal day 6–8 Balb/c mice. After decapitation, brains were removed and cut sagitally to separate the two hemispheres. Each hemisphere was embedded in physiological agar to offer mechanical support while slicing coronal sections in +3°C, modified artificial cerebrospinal fluid (aCSF) containing sucrose 195 mM, KCl 2.5 mM, NaH_2_PO_4_ 1.25 mM, NaHCO_3_ 28 mM, CaCl_2_ 0.5 mM, L-ascorbic acid 1 mM, pyruvic acid 3 mM, glucose 7 mM, and MgCl_2_ 7 mM (all from Sigma) equilibrated with 5% CO_2_ in oxygen. Sections were selected to include striatum as well as the overlying cortex. After slicing, sections were kept 15 min in ice-cold washing medium containing HBSS with HEPES 20 mM, glucose 17.5 mM, NaOH 0.88 mM and penicillin/streptomycin (all from Gibco) before placing individual slices on membrane inserts (Millipore PICM01250) in 240 µl culturing medium in 24-well dishes. The culturing medium, modified from [14] contained 50% MEM, 25% horse serum, 18% HBSS and 2% B27 supplemented with penicillin/streptomycin, glutamine 2 mM, glucose 11.8 mM, sucrose 20 mM, BDNF 30 ng/ml (R&D), GDNF 30 ng/ml (R&D) and ascorbic acid 0.2 mM (Sigma). Slices were cultured as interface cultures at 37°C, 5% CO_2_ and ambient O_2_ in 90% humidity [14], [15]. Medium was changed on day 1 of culturing and 3 times per week thereafter. B27 was withdrawn from the medium after 1 week.

**Figure 1:**
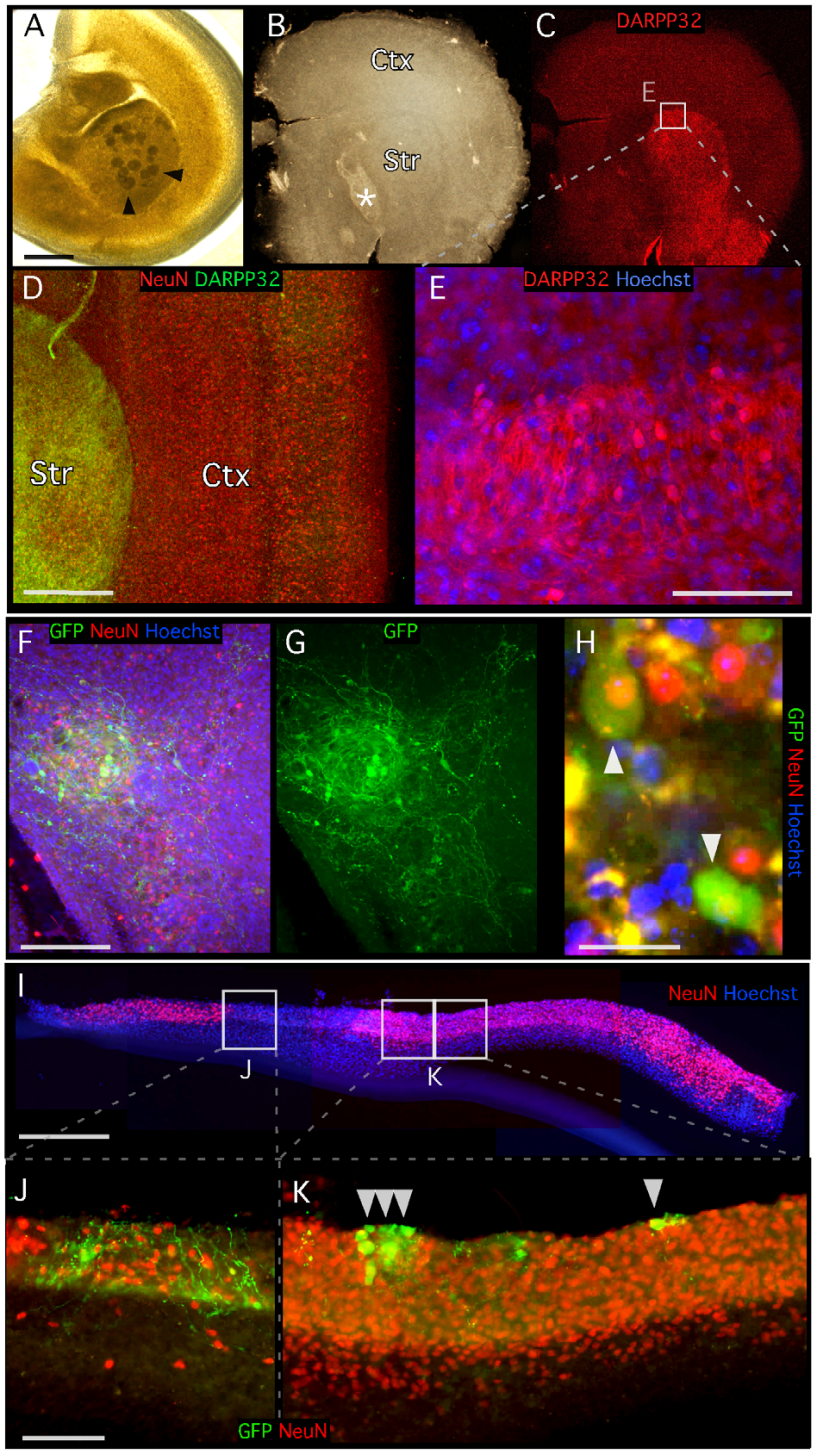
Morphology of hemisphere slice cultures and grafts. (A) One day old organotypic hemisphere culture immediately after grafting of neurospheres (arrows indicate VMN). (B, C same frame) The macro-structure of coronal hemisphere slice cultures was preserved after 3–5 weeks *in vitro*, with recognizable cortical and striatal areas and subventricular zone (Asterisk in B, cell-empty area in F, G). (C, E) DARRP32-expression was prominent in striatal cells, and confined to the striatum. (E) shows magnified area from panel (C). (B–E) DARRP32-expressing cells are clearly visible, (E) with soma and projections confined to the striatum. (D) Layered cortical cell populations remain clearly identifiable. (F, G same frame) Neurosphere integration sites in the host slices are identifiable by the GFP expression from grafted cells. Grafted dopaminergic neurons extend complex processes and migrate out of the sphere area (F, G). (H) Dopaminergic neurons in the graft express GFP, and thus TH, and the mature neuronal marker NeuN after 3–5 weeks *in vitro* (H, arrows). (I) Cross-section of a cultured slice with vertically homogenous distribution of neurons (NeuN+/Hoechst+), residing on a non-neuronal layer of cells (NeuN−/Hoechst+). The culturing membrane is seen as a blue shadow under the culture. (J) GFP-expressing fibers from grafted TH-GFP-expressing presumed dopaminergic neurons seen through all layers of the slice. (K, arrows) Grafted dopaminergic neurons residing in the upper layers of slice. Scale bars: A, B, C 1 mm; D, F, G 200 µm; E, J, K 100 µm; I 500 µm, H 25 µm.

*In vitro* grafting (co-cultures) was performed on day one after slice cultures had been started. Prior to *in vitro* grafting, VMN or VMN-*Wnt5a* cells were isolated, spun down at 800 RPM for 5 min and each (6 cm diameter) culturing dish re-suspended in 30–40 µl of organotypic slice culturing medium. Two µl re-suspension, typically containing 5–20 neurospheres, were placed on the striatal region of each slice using a standard Eppendorf pipette (see Fig. 1A). Co-culturing was done under the conditions described above for organotypic cultures.

### Optogenetic transfection of slice culture host cells

The blue light-activated depolarizing channelrhodopsin-2 (ChR2) cation channel was introduced stereotaxically into the striatum via a lentiviral construct, including the mCherry fluorescence reporter, under the CaMKIIα promoter [16], [17]. Alternatively, the hyperpolarizing orange light-driven inward chloride pump, NpHR, was delivered stereotaxically in a lentiviral construct also containing the reporter enhanced yellow fluorescent protein (YFP), under the CaMKIIα promoter [18]. Injections were performed at postnatal day 2–3, i.e., 3–4 days prior to dissections for slice cultures. Both delivery vectors were produced as described in [19] and stereotaxically injected at 2 sites (0.6 µl of titer 1–5×10^7^ per site) into the striatum of cryo-anaesthetized Balb/c mouse pups. Coordinates in relation to bregma were: anterior-posterior +0.5/0.0 mm, medio-lateral −2.0/−2.0 mm, dorsoventral −2.0/−2.0 mm.

### Optogenetic transfection of VMN cells prior to grafting

The VMN-*Wnt5a* cells were transfected with ChR2 using the same lentiviral construct as above. Two days prior to grafting onto the slice cultures, the LV-ChR2-mCherry-pCaMKIIα vector was applied directly in the culturing medium of VMN-*Wnt5a* cells at 1 infective unit per cell. The following day, transfected VMN-*Wnt5a* spheres were collected by gentle scraping, spun down at 800 RPM, and washed once in culturing medium before being transferred to new culturing medium. This was to ensure that no vector constructs were transferred to the slices during *in vitro* grafting the following day.

### Electrophysiology

After 3 weeks in co-culture, the electrophysiological properties of grafted TH-GFP positive cells in the slices were evaluated as described previously [19], [20]. Slice cultures, including neurosphere grafts, were transferred on their culturing membrane to a recording chamber continuously perfused at 4 ml/min with aCSF containing NaCl 119 mM, KCl 2.5 mM, MgSO_4_ 1.3 mM, CaCl_2_ 2.5 mM, NaHCO_3_ 26.2 mM, NaH_2_PO_4_ 1 mM, and glucose 11 mM (300 mOsm, pH 7.4; all from Sigma) at 32.5°C. For whole-cell patch-clamp recordings, a pipette solution containing K-gluconate 122.5 mM, KCl 17.5 mM, NaCl 8 mM, KOH-HEPES 10 mM, KOH-EGTA 0.2 mM, MgATP 2 mM and Na_3_GTP 0.3 mM (295 mOsm, pH 7.2; all from Sigma) was used, yielding a tip resistance of 4–5 MΩ. Biocytin was included in the pipette solution at 0.5–1 mg/ml to retrospectively identify recorded neurons. GFP-expressing cells were visualized using a wide-band excitation filter (420–480 nm), and whole-cell and patch-clamp recordings were made using infrared differential interference contrast video microscopy (BX50WI; Olympus).

Resting membrane potential (RMP) was recorded in current clamp mode at 0 pA immediately after establishing the whole-cell configuration, while postsynaptic currents were recorded in voltage clamp mode at RMP. Rectification and action potential threshold was determined from step-wise de- or hyperpolarization of the membrane potential by injecting 0.5 sec positive or negative square current pulses at 20 pA increments from RMP (0 pA). Ramp injection of 0 to 50 pA or 0 to 100 pA current over 1 sec was used to determine rheobase and action potential threshold (in addition to step depolarizations). Duration of after-hyperpolarization following action potentials was measured as the entire duration of the potential below RMP immediately following action potentials. All currents and voltages were registered and controlled using a HEKA EPC10 amplifier.

### Dopamine D_2_ receptor activation

Dopamine puffing using a Picospritzer was performed to verify the expression of DA D_2_ auto-receptors using 100 µM DA in aCSF, applied at 1–5 PSI for 10–20 sec on TH-GFP cells. After recording a steady voltage baseline at RMP, DA was applied and recordings continued for 10 sec after end of application, to verify a transient effect on D_2_ receptors. To confirm that the effect was mediated by D_2_ receptors, the D_2_ receptor antagonist Eticlopride (20 µM; Tocris Cookson) was added to the perfusion aCSF 8 min prior to DA puffing.

### GABA reversal potential

For determination of the GABA_A_ receptor activation-induced chloride current reversal potential, we used perforated patch-clamp recordings. Gramicidin A was dissolved in DMSO at 20 mg/ml and used immediately at 40 µg/ml in the intracellular solution described above. To facilitate giga-seal formation, recording pipettes were tip-filled with solution without gramicidin and backfilled with gramicidin-containing solution. After giga-seal was formed, the series resistance was monitored at regular intervals until it stabilized at 30–70 MΩ, and the integrity of the perforated patch was monitored throughout the experiment by −5 mV test pulses. To determine the GABA_A_ reversal potential of TH-GFP cells, GABA was puffed at cells using a Picospritzer (100 µM GABA in aCSF; 1–5 PSI, 5–10 ms), while membrane voltage was stepped from −20 mV to −90 mV at 10 mV increments in the presence of the respective glutamatergic NMDA and AMPA receptor blockers D-2-amino-5-phosphonopentanoate (D-AP5; 50 µM) and 2,3-dihydroxy-6-nitro-7-sulfamoyl-benzo[f]quinoxaline-2,3-dione (NBQX; 5 µM), and the voltage-gated sodium channel blocker tetrodotoxin (TTX; 2 µM) to prevent action potential firing at depolarized membrane potentials. In some recordings, the GABA_A_ antagonist picrotoxin (PTX, 100 µM) was added to block the response to GABA puffing. After recordings, slices were fixed overnight in 4% paraformaldehyde (PFA) at 4°C until further processing by immunohistochemistry.

### Optogenetic cell control

For optogenetic de- or hyperpolarization of transfected host or grafted cells, the multi-wavelength light from a standard fluorescence microscopy 2×50 W mercury burner was filtered, passing either blue light (λ420–480 nm) for ChR2 activation, or orange light (λ573–613 nm) for NpHR activation. Light was delivered via a standard 40x water immersion microscope objective, using a digitally operated shutter. Membrane current frequency was analyzed in 10 sec consecutive intervals before, during and after optical stimulation of cells.

The NpHR construct YFP reporter was reliably distinguished from the TH-GFP reporter present in grafted cells, and from mCherry from the ChR2 construct, by using different excitation filters (420–480 nm for GFP, 530–550 nm for YFP and mCherry) and emission filters (520 nm for GFP, 590 nm for YFP and mCherry). Recorded cells were additionally verified by post-hoc double-stainings for biocytin and respective reporter fluorophores.

### Lesioning and VMN transplantation *in vivo*

Lesioning of DA neurons in the substantia nigra was performed as described previously [7]. In brief, adult 25–30 g nude immuno-deficient CD-1 mice were anaesthetized with 4% chloral hydrate and injected unilaterally with 6-hydroxydopamine (6-OHDA; 3 µg, Sigma) in the right substantia nigra. Lesion severity (>90% DA cell loss) was confirmed by amphetamine-induced rotation behavior tests (>7 rotations per minute; 5 mg/kg). Ten days after lesioning, 2×2 µl of VMN-*Wnt5a*, derived from TH-GFP transgenic animals, in suspension at 100.000 cells/µl, were injected at 2 sites (from Bregma; anterior, 0.7 mm, lateral 1.75 mm, ventral 2.75 and 3.75 mm) [7]. Electrophysiology was performed at 9–10 weeks post grafting in acute 250 µm thick coronal slices of the lesioned and grafted hemisphere. Coronal slices were prepared as described for organotypic cultures, but were transferred to room temperature aCSF until electrophysiological measurements, starting 30 min after slicing.

### Immunohistochemistry

By confirming co-expression of TH-GFP and biocytin, we retrospectively validated recorded cells to be dopaminergic. Biocytin infused into cells during recordings was visualized by incubating 2 h at room temperature with Cy3-conjungated streptavidin (1∶400, Jackson) after blocking for 1 h with 5% normal horse serum at room temperature.

Immunohistochemistry was performed either on intact PFA-fixed slices attached to their culturing membranes, or on 40 µm cross-sections of these slices, still on their membranes. After an initial wash in potassium phosphate buffered saline (KPBS) and 1 h blocking in KPBS with 5% horse serum and 0.25% Triton X100, slices were incubated 15 h with antibodies against DARPP-32 (1∶200, Abcam 40801), NeuN (1∶100, Millipore MAB377), GFP (1∶10.000, Abcam 290), and mCherry (1∶1000 Chromotek 5f8) in KPBS with 1% horse serum and 0.25% Triton X100. After washing with KPBS, slices were incubated 2 h with secondary fluorophore-conjugated antibodies in KPBS with 1% horse serum and 0.25% Triton X100 to visualize reactivity of primary antibodies (Cy3 and Cy2 both 1∶300-1∶500, Jackson ImmunoResearch). Antibody specificity was confirmed by parallel stainings without the primary antibody. Counterstaining of cell nuclei was performed with Hoechst 33342 (10 ng/ml, Molecular Probes). Following immunostainings, slices and sections, still including culturing membranes, were washed with KPBS + Triton X100 and KPBS before mounting on microscopy slides using DABCO fixation.

### Statistics

Comparisons of electrophysiological properties and PCR results between groups were performed by Student's t-test. The sEPSC frequency in cells before, during and after their optical stimulation was compared with Student's paired t-test. The level of significance was set at p<0.05. All data are presented as mean ± standard error of the mean (SEM), except the GABA_A_ receptor-mediated current reversal potential, which is presented with 95% confidence interval (CI).

## Results

### Morphological characteristics of slice cultures and grafted DA neurons

The hemisphere organotypic slices were maintained in culture for 3–5 weeks without any signs of deterioration (Fig. 1A, B). Macroscopic organization, such as distinct cortical and striatal regions, was clearly identifiable (Fig. 1B). DARPP-32 expression was prominent and confined to striatal regions, with sharp borders to neighboring areas (Fig. 1C–E). NeuN immunoreactivity revealed characteristic laminar organization of neuronal populations within cerebral cortex (Fig. 1D). The subventricular zone was identified as a region deprived of cell bodies or with reduced cell body densities (Fig. 1B, F, G). Cross sectioning of the cultures revealed that slices were 40–80 µm thick. The distribution of neurons in the cross sections was uniform, though the bottom cell layer, stained with Hoechst, lacked NeuN expression (Fig. 1I).

A subset of slices was cultured for 7 months (220 days). At this time point, the organotypic macrostructure was still preserved. Hoechst staining revealed dense populations of cells though NeuN-expressing cells were less numerous, and region-specific lamination was not obvious (Fig. S2A).

*Wnt5a* applied to VMNs gives rise to a 3-fold increase of the percentage of DA neurons [7]. Therefore, we transfected the VMN cells to be used for transplantation with *Wnt5a* and found that *Wnt5a* expression one day later was 36-fold higher in the *Wnt5a*-transfected cells compared to control cells (Fig. S1). Graft-derived TH-GFP-expressing presumed DA neurons were located predominantly in the top cell layers (Fig. 1K), with processes commonly extending towards host cells in all layers of the slice cultures (Fig. 1J). *In vitro* grafted spheres were visible immediately after transplantation (Fig. 1A) but were indiscernible in the host tissue less than an hour later, and subsequently recognizable only by the expression of TH-GFP (Fig. 1B, K). Dopaminergic neuron density was generally higher at the engraftment border of the donor sphere (Fig. 1F, G, K), though individual TH-GFP expressing neurons were commonly observed at hundreds of µms from identifiable engraftment sites, indicating migration of these cells within the host tissue (Fig. 1G, K, single arrowhead). Neurosphere engraftment sites varied in size, from large with tens of GFP-expressing cells (Fig. 1F, G) to small with only few GFP neurons (Fig. 1K, triple arrowheads). The GFP-expressing processes formed complex networks that extended several 100 µm from the cell bodies, seemingly innervating other cells both in the graft and host tissue (Fig. 1F, G, J).

After 3–5 weeks *in vitro*, TH-GFP-expressing, presumed DA neurons on the surface of the slices co-expressed NeuN and commonly appeared round and flat with relatively weak auto-fluorescence (Fig. 1H arrowheads). These characteristics were maintained in slices cultured for 7 months (Fig. S2C).

### Electrophysiological characteristics of grafted DA neurons

**Figure 2:**
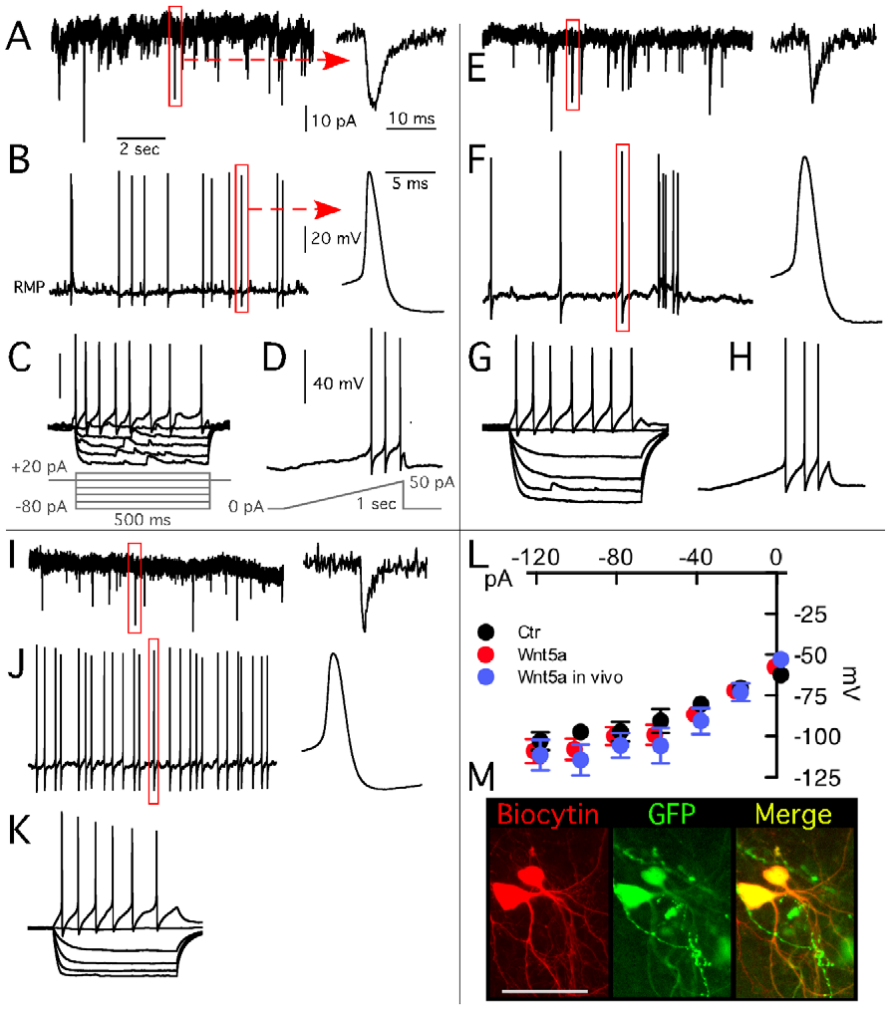
Intrinsic electrophysiological properties of control and *Wnt5a*-transfected grafted cells *in vitro* and *in vivo.* (A–D) sEPSCs and action potentials recorded in *Wnt5a* non-transfected grafted cells in organotypic cultures. (E–H) sEPSCs and action potentials recorded in *Wnt5a* transfected grafted cells in organotypic cultures. (I–K) sEPSCs and action potentials recorded in *Wnt5a* transfected grafted cells *in vivo* in acute slices. (L) Averaged I/V curves from all three groups. All three groups included spontaneously firing cells, with relatively broad action potentials, typical of dopaminergic neurons (B, F, J). For co-cultures the action potential threshold was determined both from spontaneously firing cells and from current ramps (D, H). The rheobase was also determined from these ramps. Averaged data for all three groups and their comparisons are presented in Table S1. (M) Two recorded cells exemplifying documentation of TH-GFP genotype retrospectively confirmed by infused biotcytin into cells during recordings.

**Figure 3:**
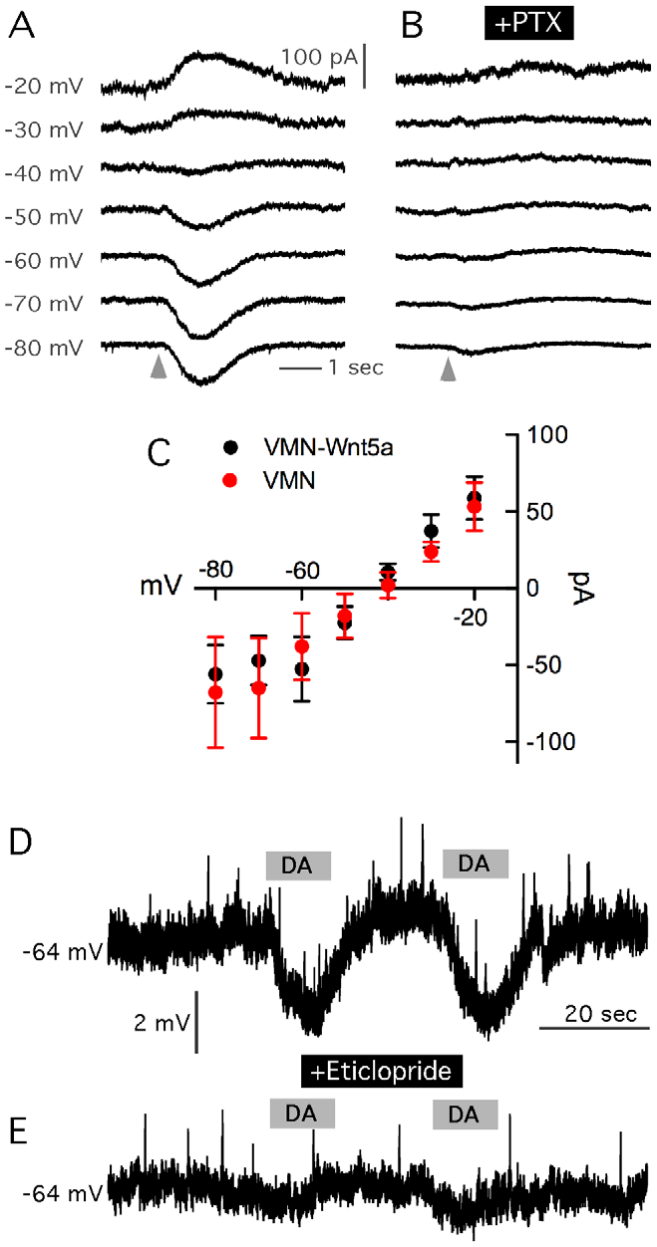
Reversal potential of GABA_A_ receptor-mediated currents and responses to DA of graft-derived dopaminergic neurons. (A) Perforated patch-clamp recording of grafted VMN neuron during extracellular application of GABA (with Picospritzer, grey arrows) at various holding membrane potentials. (B) The GABA_A_ antagonist PTX blocked the response to GABA application. (C) Averaged responses to GABA application at various holding membrane potentials. Note relatively high reversal potential of GABA responses in both VMN (−42.3 mV; n = 7) and VMN-*Wnt5a* (−44.8; n = 7) cells. (D) Recording of grafted VMN cell during extracellular application (with Picospritzer, grey lines) of DA. DA effected a 4–7 mV hyperpolarization of the membrane potential in VMN (n = 6) and VMN-*Wnt5a* (n = 6). (E) DA responses were blocked by pretreatment of slices with D_2_-receptor antagonist Eticlopride. Averaged data from these experiments are presented in Table S1.

*Wnt5a* expression did not influence the intrinsic membrane properties of the grafted VMN-derived DA cells in the organotypic slice co-cultures (for details see Text S1 and Table S1). Most properties resembled those of DA neurons in the intact substantia nigra [21]. However, none of the grafted DA cells expressed delayed rectification activated by hyperpolarizing pulses, so-called “sag” (Fig. 2C, G, K), which is a characteristic feature of intrinsic DA neurons [22]. To further explore the synaptic and receptor profile of the grafted DA cells, we tested the expression of functional GABA receptors in organotypic cultures by local puffing of 100 µM GABA while recording from TH-GFP neurons with the perforated patch-clamp technique. This was performed in the presence of blockers of glutamatergic transmission, D-AP5 (50 µM) and NBQX (5 µM), and the voltage-gated sodium channel blocker TTX (2 µM). When varying holding membrane potentials of the cells from −20 mV to −90 mV during GABA application, the GABA reversal potential in the VMN*-Wnt5a*-derived TH-GFP cells was −42.3 mV (95% CI: −49.75 to −32.18, n = 7; in VMN control cells −44.8 mV; 95% CI: −49.89 to -39.10, n = 7; see Fig. 3C). The GABA-activated currents were mediated by GABA_A_ receptors, since they were blocked by application of 100 µM picrotoxin (Fig. 3A, B). Only one perforated patch-clamp recording was possible to obtain from a VMN-*Wnt5a*- derived DA neuron after 7 months of organotypic culturing, with an estimated GABA reversal potential of −49.4 mV (Line of best fit, 95% CI: −50.9 to −47.8).

We then assessed the expression of DA D_2_ receptors on grafted TH-GFP positive cells in organotypic cultures by local puffing of 100 µM DA for 10 to 20 seconds. This induced membrane hyperpolarization both in VMN-*Wnt5a*- (−6.1±0.9 mV; n = 6; Fig. 3D) and VMN-derived DA cells (−4.7±1.0 mV; n = 6). The hyperpolarizing effect of DA was antagonized by application of the D_2_ receptor antagonist Eticlopride (20 µM) in all recorded cells, indicating that the effect was mediated via D_2_ receptors (Fig. 3E). Puffing of aCSF alone never induced membrane voltage changes (data not shown).

To determine whether the morphological and electrophysiological properties of grafted cells *in vitro* resembled those *in vivo*, VMN-*Wnt5a* cells were transplanted into the DA-depleted striatum of 6-OHDA-lesioned animals. Ten weeks after transplantation, TH-GFP-expressing, presumed DA neurons were observed mostly along the injection tract (Fig. S3A, B). Biocytin infused into cells during patch-clamp recordings in acute slices revealed that processes of these cells were largely confined to the injection tract (Fig. S3B). Electrophysiologically, *in vivo* grafted VMN-*Wnt5a* DA neurons were remarkably similar to those grafted *in vitro*, displaying mature functional properties and no delayed inward rectification (Fig. 2I–L, Text S1; Table S1).

### Functional inputs from host to grafted stem cell-derived DA neurons

**Figure 4:**
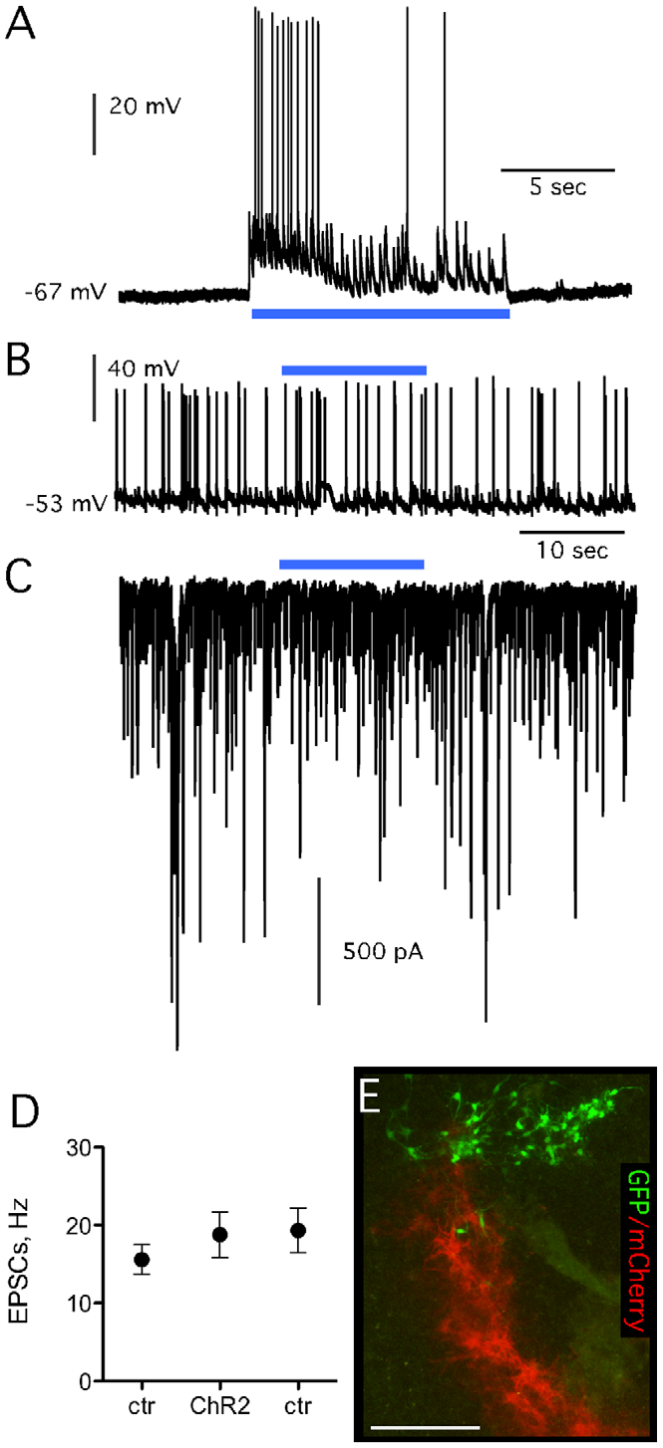
Host-to-graft synaptic connectivity in organotypic slice cultures probed with ChR2. (A) Striatal ChR2-transduced host neurons in organotypic slice cultures responded to blue light illumination with immediate depolarization of the membrane potential and action potential generation. (B, C) Activation of host striatal neurons did not elicit any detectable response in grafted VMN-*Wnt5a*-derived GFP expressing neurons, neither in voltage-clamp (C) nor in current-clamp (B) modes. (D) Averaged frequency of sEPSCs recorded before, after [controls; (ctr)] or during blue light illumination (ChR2). (E) Microphotograph depicting relatively close locations of recorded TH-GFP cells and ChR2-mCherry expressing host cells.

Two optogenetic approaches were used. In the first approach, ChR2-transduced host cells were activated by blue light while we recorded activity in grafted GFP-expressing DA neurons derived from *Wnt5a*-expressing VMN cells. The mCherry reporter included in the vector construct confirmed expression of the ChR2 transgene under the CaMKIIα promoter in host striatal neurons in the area of the vector injection site (Fig. 4E). Upon exposure to blue light, an instant depolarization of transduced host striatal neurons was observed, lasting exactly for the duration of light exposure (Fig. 4A). The light-induced depolarization triggered action potential firing in two of four recorded transduced host cells. No detectable response was observed in grafted DA neurons while transduced host striatal cells were activated by light. Basal sEPSC frequency in grafted cells before, during, and after light activation of host ChR2 transduced cells was 15.6±1.9, 18.8±2.9, and 19.4±2.9 Hz (n = 10; Fig 4B–D).

**Figure 5:**
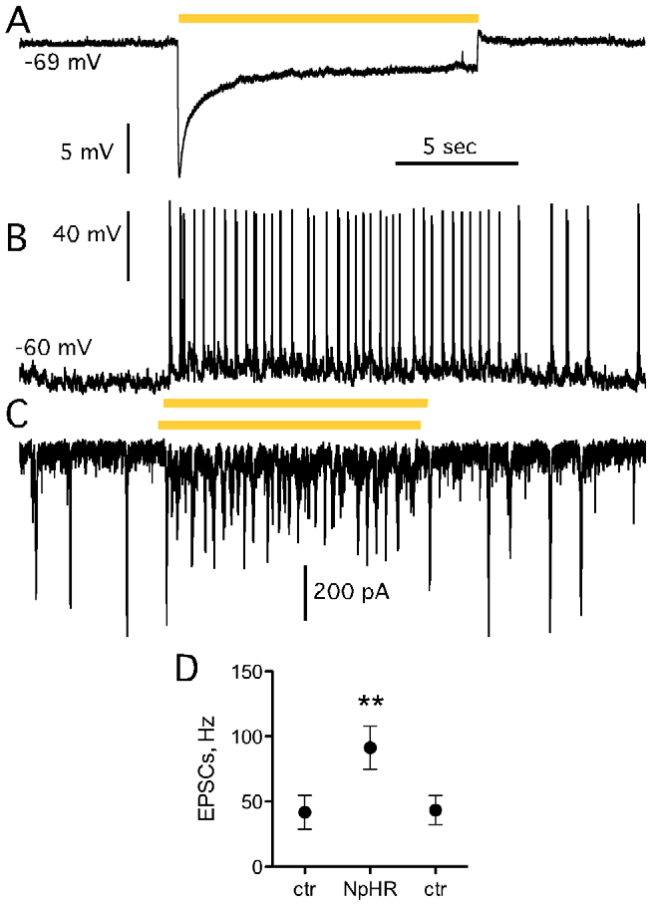
Host-to-graft synaptic connectivity in organotypic slice cultures probed with NpHR. (A) Striatal NpHR-transduced host neurons in organotypic slice cultures responded to orange light illumination with immediate hyperpolarization of the membrane potential. (B, C) Increased frequency of sEPSCs (C) accompanied with increased frequency of action potential firing (B) of grafted dopaminergic neurons, when host striatal neurons were silenced by orange light illumination. (D) Averaged frequencies of sEPSCs (≥50 pA amplitude) in grafted DAergic neurons before and after (ctr) or during (NpHR) orange light illumination.

In the second optogenetic approach, NpHR-transduced host striatal neurons were silenced through orange light illumination while recording from grafted DA neurons. Striatal NpHR expression was driven by the CaMKIIα promoter and assessed by visualizing YFP included in the viral vector construct as a reporter. Upon exposure to orange light, NpHR activation induced inward chloride pumping and immediately hyperpolarized host striatal neurons by 5–10 mV (n = 3; Fig. 5A). Whole-cell recordings from grafted DA neurons revealed increased frequency of EPSCs upon silencing of host cells from 37.4±12.0 Hz before NpHR activation to 81.6±17.3 Hz, which returned to 39.5±10.3 Hz immediately when illumination was stopped (Fig. 5C–D; n = 5). In two out of five recorded cells, this increase in sEPSCs was accompanied by increased action potential frequency (Fig. 5B). No grafted TH-GFP-expressing DA cells were ever transduced by the lentiviral vectors applied to the organotypic cultures 4–5 days prior to grafting (Fig. 4E).

### Functional outputs from grafted VMN-derived DA neurons to the host

**Figure 6:**
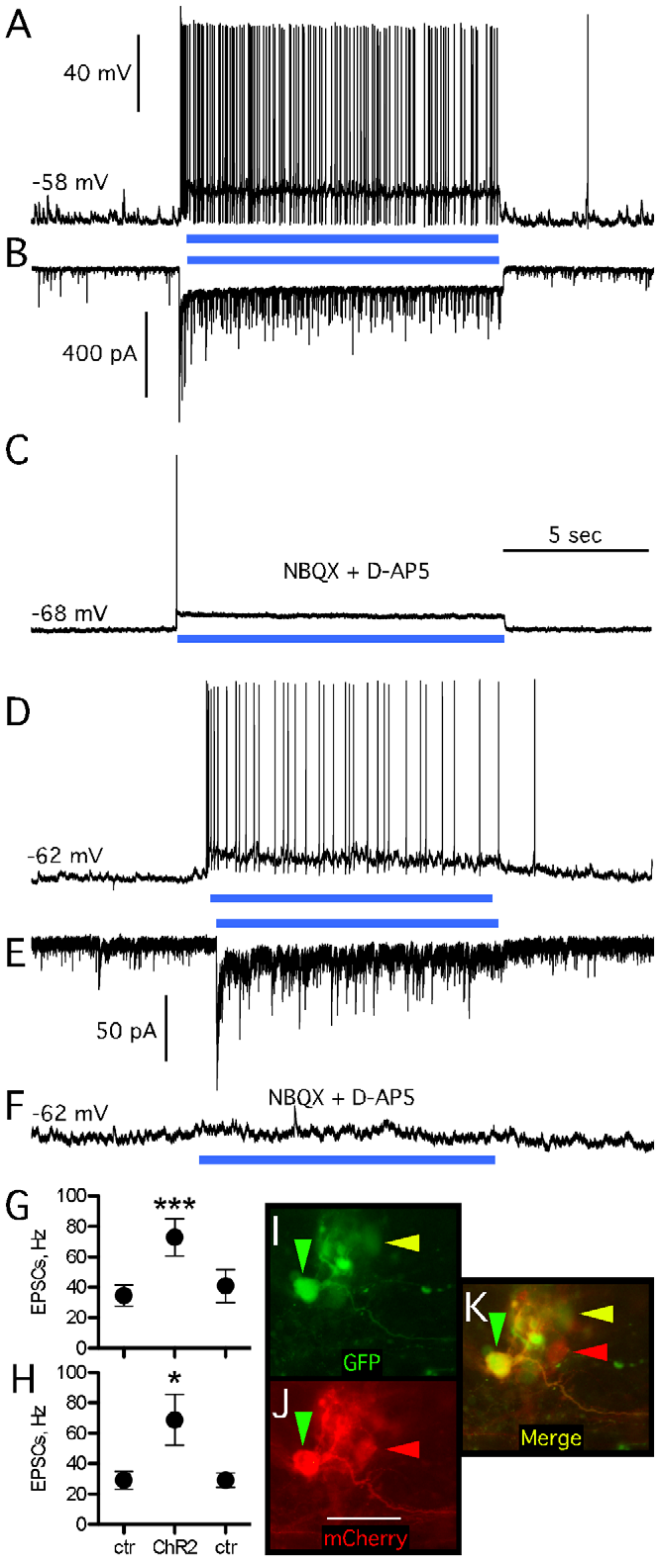
Intragraft synaptic connectivity in organotypic slice cultures probed by ChR2. (A, B) Grafted cells, including GFP-expressing presumed dopaminergic neurons transduced by ChR2 responding with depolarization and action potentials to blue light illumination. (C) Blue light illumination-induced depolarization of ChR2-expressing grafted neuron during block of excitatory inputs from other neighboring ChR2-expressing grafted cells. (D, F) Blue light illumination-induced response in current-clamp mode (D) and voltage-clamp mode (E) of grafted neuron not expressing ChR2: In this cell blockade of excitatory inputs from other neighboring ChR2-expressing grafted cells completely eliminated light-induced response (F). Note scale difference between (B) and (E). (G, H) Averaged frequency of sEPSCs in ChR2-transduced and non-transduced TH-GFP-expressing grafted neurons before and after (ctr), and during (ChR2) blue light illumination. Note similar increase of sEPSC frequencies induced by blue light illumination in both populations of grafted neurons. (I, K green arrow) ChR2-transduced and (I, K yellow arrow) non-transduced presumed dopaminergic TH-GFP neurons, as well as (J, K red arrow) ChR2-transduced mCherry expressing non-TH-GFP cells, in the same graft site.

**Figure 7:**
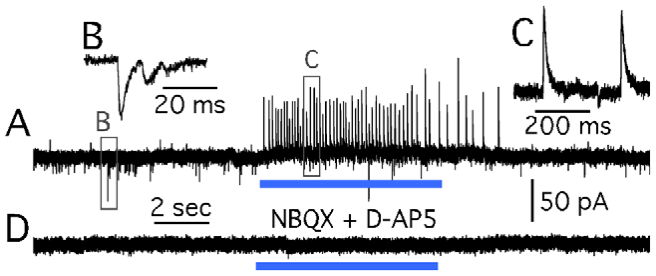
Graft-to-host synaptic connectivity in organotypic slice cultures probed with ChR2. (A, C) Host cell responding to selective ChR2 activation of grafted cells with an increase in large amplitude outward (hyperpolarizing) currents most likely resembling GABA_A_ receptor mediated sIPSCs. (B) Inward (depolarizing) currents most likely resembling sEPSCs. (D) The response to light illumination blocked by antagonists of glutamatergic signaling, suggesting a poly-synaptic nature of the response. Outward hyperpolarizing currents were never observed in grafted cell recordings.

The CaMKIIα-ChR2-mCherry construct was successfully expressed in the stem cell-derived neurons by exposing these cells to the lentiviral vector *in vitro* prior to grafting (Fig. 6I–K). Upon illumination by blue light, the grafted ChR2-transduced neurons in the organotypic cultures were immediately depolarized (Fig. 6A–E). This was followed by an immediate increase in frequency of sIPSCs in only one out of six recorded host neurons lasting exactly the same time as the light exposure (Fig. 7A, C). Application of NBQX and D-AP5 blocked the response of host cells to graft activation, showing that these sIPSCs were not monosynaptic (Fig. 7D). The frequency of sEPSCs in the host cells was not affected by the light (Fig. 7A–C).

### Functional connections between neurons within the graft

To investigate intragraft synaptic connectivity, VMN*-Wnt5a*-derived DA neurons were recorded, while all grafted ChR2-expressing cells were optically activated. Retrospective immunocytochemical staining showed that grafted, presumed DA cells expressed both GFP and mCherry, or GFP only, while other grafted cells expressed mCherry only (Fig. 6I–K). All recorded graft-derived DA neurons responded to light-stimulation by increasing sEPSC and action potential firing frequency (Fig. 6A–H). This was the case for both ChR2-transduced (n = 5 neurons, Fig. 6A–C), and ChR2 non-transduced (n = 4 neurons, Fig. 6D–F) DA neurons. Since generation of action potentials could have also resulted from optogenetic activation of the recorded ChR2-transduced grafted DAergic neurons, we only analyzed frequencies of sEPSCs during blue light illumination. In ChR2-transduced DA neurons, the frequency of sEPSCs immediately before and after ChR2 activation was 34.6±6.9 and 42.0±10.9 Hz, respectively, while it increased to 72.9±12.2 Hz during ChR2 activation (Fig. 6G).

Intragraft excitation could be mediated by graft-derived non-dopaminergic glutamatergic cells, though it is also possible that graft-derived DA neurons, which have also been shown to release glutamate [23], are contributing to increased sEPSC frequency in recorded DA neurons. Moreover, glutamatergic autapses of recorded DA neurons could potentially contribute to increased sEPSC frequency [24], [25]. However, since similar increase in sEPSC frequency during illumination was observed in DA neurons not transduced by ChR2 (Fig. 6H), the latter scenario is unlikely. Spontaneous IPSCs were not observed during recordings of any grafted cells (e.g. Fig. 6A, E).

## Discussion

Here we have applied, for the first time, optogenetic methods in combination with patch-clamp recordings to analyze the functional integration of stem cell-derived DA neurons grafted into an *in vitro* PD model. We demonstrate complex bidirectional functional interaction between grafted DA neurons and host cells and extensive intragraft excitatory synaptic connectivity.

Our findings indicate that the organotypic hemisphere cultures used here, having relatively preserved cortico-striatal connections but severed DA input to the striatum, represent a suitable model for preliminary screening of the functional properties of stem cell-derived DA neurons and their integration into host neural circuitries. Most hallmarks of mesencephalic DA neurons *in situ* were detected in the stem cell-derived DA cells, including presynaptic D_2_ autoreceptors [26]. However, the characteristic delayed inward rectifying “sag” was not observed in any of the grafted DA neurons [27]. Since grafted stem cell-derived DA neurons lacking “sag” have been shown to ameliorate functional deficits in 6-OHDA lesioned animals [7], the significance of the delayed inward rectifier currents for the therapeutic action of grafted DAergic neurons is unclear. We also found, using perforated patch-clamp recordings in combination with puff application of GABA, that the reversal potential for GABA_A_ receptor-mediated chloride currents in the DA neurons was near −40 mV. Such depolarized reversal potential of GABA_A_ receptor-mediated currents seems to be characteristic of endogenous DA neurons [28], but may also indicate partial immaturity [29], [30]. To our knowledge, this is the first time that the reversal potential of GABAergic currents has been explored in grafted DA neurons.

Observations in PD patients subjected to intrastriatal transplantation of fetal VM tissue indicate that the grafted DA neurons become functionally integrated into host neural circuitries [31], a process which parallels the time course of clinical improvement. However, due to their ectopic location, intrastriatally grafted DA neurons do not receive “correct” synaptic inputs [32]. Exploring the functional synaptic interactions between grafted stem cell-derived DA cells and host neurons in experimental models is not trivial and has been hindered due to technical limitations. Kim et al. [5] performed paired electrophysiological recordings from presumed striatal host cells and grafted mouse ES-derived DA neurons. No direct synaptic interactions between these pairs were found [5]. Electrical field stimulations within the graft area induced EPSCs in both graft and presumed host neurons [5]. The host identity of neurons was inferred solely by their distance from the graft site. In acute slices, field stimulations presumably outside the transplant have been shown to induce both glutamatergic and GABAergic postsynaptic currents in grafted fetal DA neurons [33], as well as in grafted neural stem cell-derived neurons [34]. When measuring extracellularly from presumed grafted fetal mesencephalic neurons *in vivo*, field stimulations in both the striatum and cortex evoked increased action potential firing in some neurons, while in the majority of grafted neurons such stimulations decreased the action potential firing rate, both suggesting host-to-graft connectivity [35]. However, in none of the above studies the source of synaptic inputs to the graft neurons was unequivocally verified to be host-derived.

We demonstrate here that the optogenetic approach makes possible completely selective activation and inhibition of either host or grafted neurons in an unprecedented manner. In our experiments, action potentials induced by light in the ChR2-transduced host striatal neurons did not reveal any direct synaptic connections to the grafted DA cells. Interestingly, silencing of the host striatal, NpHR-transduced neurons by selective light activation increased excitatory synaptic activity in the grafted cells. One possible explanation to this observation is that the grafted neurons receive direct cortical excitatory inputs, which are presynaptically tonically suppressed by inhibitory striatal neuron collaterals, similar to what has been described for cortical innervation of host striatal neurons *in vivo* [36], [37]. Selective silencing of these striatal neurons by optogenetic approach results in a disinhibitory effect, revealed as increased excitatory cortical input to the grafted cells. Optogenetic activation of grafted DA and other neurons resulted in appearance of GABA_A_ receptor-mediated slow IPSCs only in one out of six recorded host neurons. This connection was most likely polysynaptic since application of glutamatergic receptor antagonists blocked the response. Similar optogenetic activation of grafted neurons always induced increased sEPSC frequency in grafted DA neurons, indicating extensive intragraft excitatory connections.

Taken together, our data indicate that in the *in vitro* PD model, there is extensive synaptic interaction between DA neurons and other cell types within the graft and that connections between host and graft are complex, including host striatal presynaptic regulation of host cortical afferents to the grafted DA neurons and polysynaptic graft-to-host connections. Further optogenetic dissection of the functional synaptic interactions between grafted and host neurons will be instrumental in delineating the cellular and synaptic mechanisms underlying behavioral recovery, as well as those which may give rise to adverse effects [38] following DA cell transplantation in patients with Parkinson's disease.

## Supporting Information

Figure S1 **Increased** ***Wnt5a*** **expression 1 day after plasmid transfection.** Q-PCR results, comparing *Wnt5a* expression to that of control pCAIP-transfected cells. *Wnt5a* transfection increased the expression to 35.9±8.3 when normalized to empty plasmid control transfections at 1.0±0.14 (both n = 4).
(TIF)

Figure S2 **Organotypic cultures after 7 months of culturing.** (A) Organotypic hemisphere cultures were densely populated by cells as assessed by Hoechst staining, though there seemed to be fewer NeuN expressing cells as compared to 3–5 weeks time point. (B, C) VMN-*Wnt5a*-derived GFP expressing neurons, morphologically identical to those at 3–5 weeks of culturing. Note GFP-expressing cells positive to NeuN (nuclei; C). (A) and (B) depict same frame. (C) is a magnified from B. Scale bars: A 100 µm; B 50 µm. (D–G) depicts electrophysiological properties of representative graft-derived DA neurons after 7 months *in vitro*. They largely resembled those of the cells at 3–5 weeks in vitro, including spontaneous firing of action potentials (D), presence of excitatory postsynaptic currents, and complete lack of inhibitory postsynaptic currents (E, F). Delayed rectification, *sag*, was still not present at this stage (G).
(TIF)

Figure S3 **Grafted VMN** ***-Wnt5a*** **-derived dopaminergic neurons** ***in vivo.*** (A) VMN-*Wnt5a* neuron expressing TH-GFP 10 weeks after grafting *in vivo* into the DA-depleted mouse striatum. (B) magnified square in (A). Note that GFP-expressing cells were predominantly observed in, or immediately around, the injection tract (A, B). Biocytin-filled cells revealed processes from GFP-expressing presumed dopaminergic neurons mostly confined to the injection tract (B). Scale bars: A 100 µm; B 50 µm.
(TIF)

Table S1 **Intrinsic electrophysiological properties.** Intrinsic membrane properties of VMN and VMN-*Wnt5a* cells grafted into striatal slice cultures, and of VMN-*Wnt5a* cells grafted into striatum of 6-OHDA lesioned mice. Note no differences in parameters between VMN and VMN-*Wnt5a* cells in striatal slice cultures after 3–5 weeks *in vitro*. Last column represents electrophysiological properties of VMN-*Wnt5a*-derived GFP expressing neurons after 7 months in organotypic slice cultures. Comparisons were made between VMN and VMN-*Wnt5a* TH-GFP cells at 3–5 weeks *in vitro*, between both these groups and VMN-*Wnt5a in vivo* measured in acute slices, and between VMN-*Wnt5a* at 3–5 weeks and at 7 months *in vitro*. * p<0.05 relative to VMN-*Wnt5a in vivo*; ^Δ^ p<0.05 relative to VMN-*Wnt5a* after 3 weeks; • n = 7, 8, 6; •• n = 6, 6. See supporting results for details (Text S1).
(DOC)

Text S1 **Supporting Results.**
(DOC)
