# Supplementary figures and images for: Functional Integration of Grafted Neural Stem Cell-Derived Dopaminergic Neurons Monitored by Optogenetics in an In Vitro Parkinson Model
Source: PLoS One. 2011 Mar 4;6(3):e17560. doi: 10.1371/journal.pone.0017560 (PMC3048875; doi:10.1371/journal.pone.0017560)

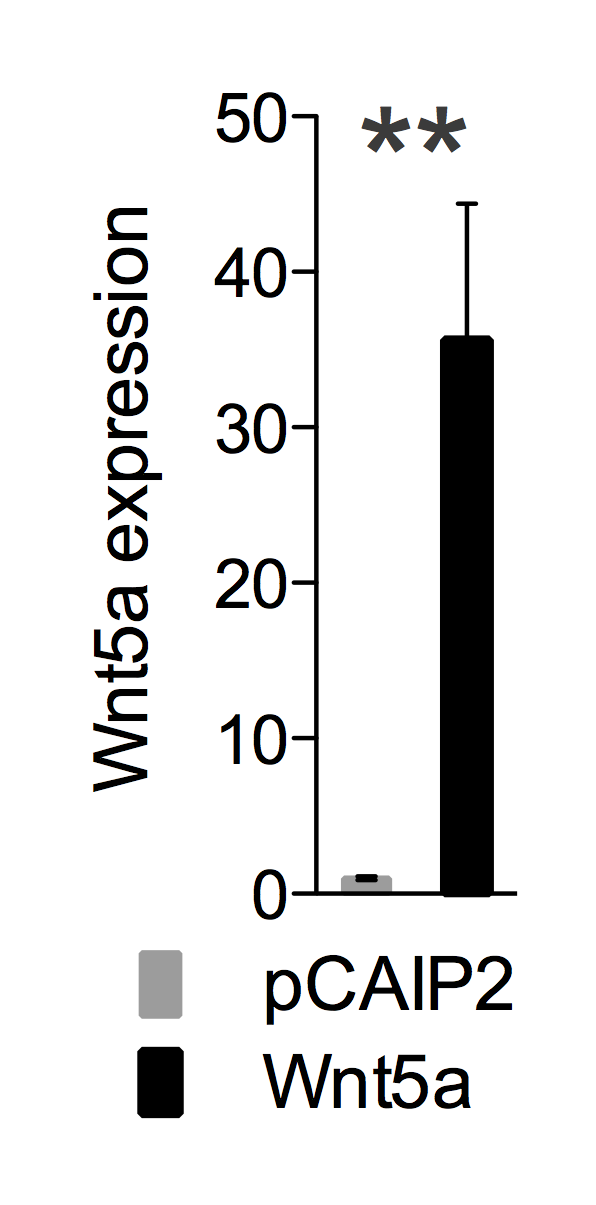

Supplement: Figure S1 — Increased Wnt5a expression 1 day after plasmid transfection. Q-PCR results, comparing Wnt5a expression to that of control pCAIP-transfected cells. Wnt5a transfection increased the expression to 35.9±8.3 when normalized to empty plasmid control transfections at 1.0±0.14 (both n = 4). (TIF) [file pone.0017560.s001.tif]

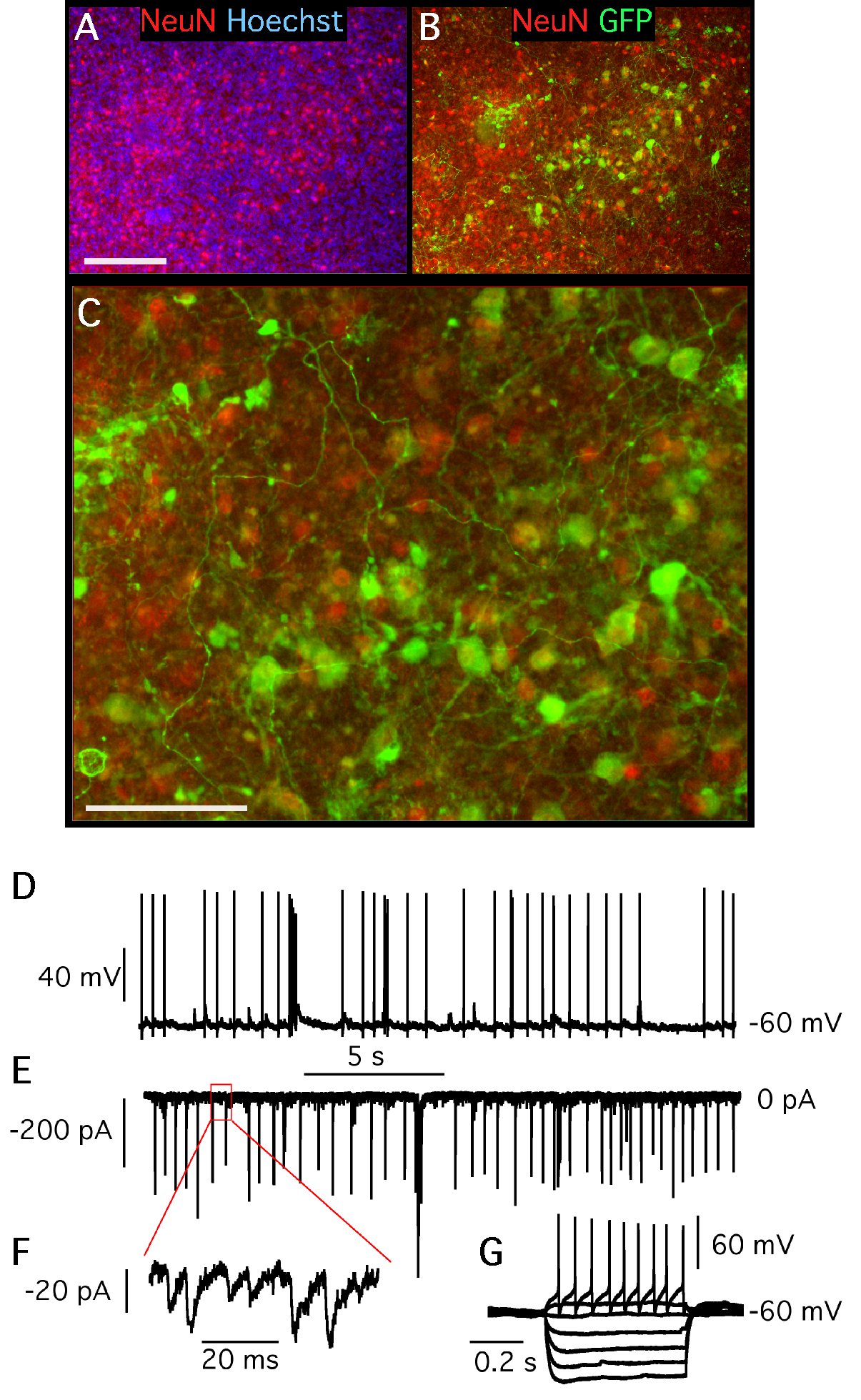

Supplement: Figure S2 — Organotypic cultures after 7 months of culturing. (A) Organotypic hemisphere cultures were densely populated by cells as assessed by Hoechst staining, though there seemed to be fewer NeuN expressing cells as compared to 3–5 weeks time point. (B, C) VMN-Wnt5a-derived GFP expressing neurons, morphologically identical to those at 3–5 weeks of culturing. Note GFP-expressing cells positive to NeuN (nuclei; C). (A) and (B) depict same frame. (C) is a magnified from B. Scale bars: A 100 µm; B 50 µm. (D–G) depicts electrophysiological properties of representative graft-derived DA neurons after 7 months in vitro. They largely resembled those of the cells at 3–5 weeks in vitro, including spontaneous firing of action potentials (D), presence of excitatory postsynaptic currents, and complete lack of inhibitory postsynaptic currents (E, F). Delayed rectification, sag, was still not present at this stage (G). (TIF) [file pone.0017560.s002.tif]

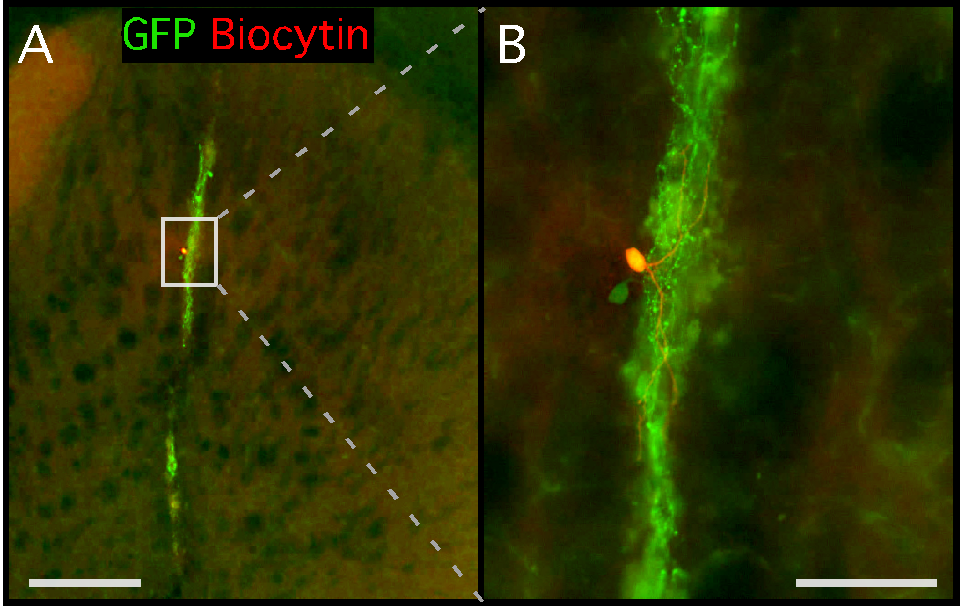

Supplement: Figure S3 — Grafted VMN -Wnt5a -derived dopaminergic neurons in vivo. (A) VMN-Wnt5a neuron expressing TH-GFP 10 weeks after grafting in vivo into the DA-depleted mouse striatum. (B) magnified square in (A). Note that GFP-expressing cells were predominantly observed in, or immediately around, the injection tract (A, B). Biocytin-filled cells revealed processes from GFP-expressing presumed dopaminergic neurons mostly confined to the injection tract (B). Scale bars: A 100 µm; B 50 µm. (TIF) [file pone.0017560.s003.tif]
