# Supplementary material for: Functional Integration of Grafted Neural Stem Cell-Derived Dopaminergic Neurons Monitored by Optogenetics in an In Vitro Parkinson Model
Source: PLoS One. 2011 Mar 4;6(3):e17560. doi: 10.1371/journal.pone.0017560 (PMC3048875; doi:10.1371/journal.pone.0017560)
Supplement: Table S1 — Intrinsic electrophysiological properties. Intrinsic membrane properties of VMN and VMN-Wnt5a cells grafted into striatal slice cultures, and of VMN-Wnt5a cells grafted into striatum of 6-OHDA lesioned mice. Note no differences in parameters between VMN and VMN-Wnt5a cells in striatal slice cultures after 3–5 weeks in vitro. Last column represents electrophysiological properties of VMN-Wnt5a-derived GFP expressing neurons after 7 months in organotypic slice cultures. Comparisons were made between VMN and VMN-Wnt5a TH-GFP cells at 3–5 weeks in vitro, between both these groups and VMN-Wnt5a in vivo measured in acute slices, and between VMN-Wnt5a at 3–5 weeks and at 7 months in vitro. * p<0.05 relative to VMN-Wnt5a in vivo; Δ p<0.05 relative to VMN-Wnt5a after 3 weeks; • n = 7, 8, 6; •• n = 6, 6. See supporting results for details (Text S1). (DOC) [file pone.0017560.s004.doc]

|  | **In vivo (10 weeks)** | | | **Co-culture (3-5 weeks)** | | | | | | **(7 months)** | | |
| --- | --- | --- | --- | --- | --- | --- | --- | --- | --- | --- | --- | --- |
|  | **VMN-Wnt5a, N=9** | | | **VMN, N = 9** | | | **VMN-Wnt5a, N = 8** | | | **VMN-Wnt5a, N = 6** | | |
| **Rinp, MΩ** | 1039 | ± | 113 | 609.0 | ± | 97.5 * | 678.5 | ± | 102.7 * | 582.2 | ± | 91.6 |
| **RMP, mV** | -54 | ± | 2.5 | -60.4 | ± | 2.8 | -56.4 | ± | 3.1 | -59.9 | ± | 3.0 |
| **Action Potential** |  |  |  |  |  |  |  |  |  |  |  |  |
| **Spontaneous APs, cells** | 7 of 9 | | | 7 of 9 | | | 4 of 8 | | | 5 of 6 | | |
| **Frequency, Hz** | 4.9 | ± | 1.5 | 1.3 | ± | 0.7 | 1.3 | ± | 0.9 | 2.3 | ± | 0.7 |
| **Threshold, mV, step** | -42.9 | ± | 1.2 | -38.6 | ± | 1.5 * | -39.3 | ± | 1.0 * | -33.3 | ± | 1.4^∆^ |
| **Threshold, mV, ramp •** |  |  |  | -41.3 | ± | 0.9 | -39.4 | ± | 1.8 | -34.8 | ± | 1.6 |
| **Rheobase, pA •** |  |  |  | 36.5 | ± | 5.7 | 39.4 | ± | 7.3 | 88.5 | ± | 39.2 |
| **Amplitude, mV** | 80.1 | ± | 3.2 | 78.8 | ± | 3.0 | 73.5 | ± | 2.9 | 82.2 | ± | 3.9 |
| **Duration, ms** | 1.9 | ± | 0.1 | 1.6 | ± | 0.2 | 1.6 | ± | 0.1 | 0.8 | ± | 0.1^∆^ |
| **After-hyperpolarization** |  |  |  |  |  |  |  |  |  |  |  |  |
| **Amplitude, mV** | 9.7 | ± | 1.3 | 13.4 | ± | 2.1 | 15.0 | ± | 1.7 * | 13.9 | ± | 1.8 |
| **Duration, ms** | 47.1 | ± | 6.4 | 17.8 | ± | 7.2 * | 22.9 | ± | 5.3 * | 14.4 | ± | 3.0 |
| **D2 response, mV ••** |  |  |  | -4.7 | ± | 1.0 | -6.1 | ± | 0.9 |  |  |  |
