# Supplementary material for: Functional Integration of Grafted Neural Stem Cell-Derived Dopaminergic Neurons Monitored by Optogenetics in an In Vitro Parkinson Model
Source: PLoS One. 2011 Mar 4;6(3):e17560. doi: 10.1371/journal.pone.0017560 (PMC3048875; doi:10.1371/journal.pone.0017560)
Supplement: Text S1 — Supporting Results. (DOC) [file pone.0017560.s005.doc]

**Text S1**

**Supporting Results**

Series resistances in VMN and VMN-*Wnt5a*-derived DA neurons were 19.4 ± 2.0 and 22.2 ± 1.2 MΩ, respectively, and the corresponding input resistances were 609.0 ± 97.5 and 678.5 ± 102.7 MΩ. VMN and VMN-*Wnt5a*-derived DA neurons had resting membrane potential (RMP) of -60.4 ± 2.8 and -56.4 ± 3.1, respectively. Spontaneous firing, defined as one or more action potentials in the initial 30 seconds of recording at RMP (0 pA current clamp), was observed in seven of nine control VMN-derived DA neurons, and in four of eight VMN-*Wnt5a*-derived DA neurons (Fig. 2B, F). Action potential threshold was identical for VMN and VMN-*Wnt5a*-derived DA neurons, being -38.6 ± 1.5 mV and -39.3 ± 1.0 mV. The thresholds were similar when determined either by applying depolarizing current ramps (Fig. 2D, H), or current steps (Fig. 2C, G; Table S1). For both groups, action potential duration was 1.6 ms measured at half peak amplitude, and the amplitudes were not different between the groups (78.8 ± 3.0 mV for VMN; Fig. 2B; and 73.5 ± 2.9 mV for VMN-*Wnt5a*; Fig. 2F). The after-hyperpolarization amplitude following action potentials was similar in VMN and VMN*-Wnt5a* (13.4 ± 2.1 mV and 15.0 ± 1.7 mV, respectively), as was the after-hyperpolarization duration (17.8 ± 7.2 ms and 22.9 ± 5.3 ms, respectively). Inward rectification of the membrane was not different between VMN and VMN-*Wnt5a*-derived DA neurons when assessed by hyperpolarizing current steps up to -120 pA, resulting in a membrane hyperpolarization to near -110 mV (Fig. 2L). Delayed rectification “sag” was not observed in any of the recorded cells in both groups (Fig. 2C, G, L).

For further validation of neuronal properties, the electrophysiological characteristics of grafted VMN-*Wnt5a*-derived DA neurons were also studied in acute hemisphere slices derived from 6-OHDA lesioned animals 10 weeks after transplantation. The intrinsic functional properties of these cells were mostly similar to that of VMN-*Wnt5a*-derived DA neurons differentiated in organotypic cultures (Table S1). For example, action potential duration and amplitude (Fig. 2J), as well as inward rectification (Fig. 2L) were similar to those in cells differentiated in organotypic cultures. However, the action potential threshold as estimated by step depolarizations was higher in *in vivo* grafted cells (-42.9 ± 1.2 mV; Fig. 2K), as compared to both groups in organotypic cultures (Table S1). Moreover, action potential after-hyperpolarization was lower in amplitude but longer in duration in VMN-*Wnt5a* grafted *in vivo* (9.7 ± 1.3 mV; 47.1 ± 1.0 ms, respectively) as compared to those in organotypic cultures (VMN: 13.4 ± 2.1 mV and 17.8 ± 7.2 ms; VMN-*Wnt5a*: 15.0 ± 1.7 mV and 22.9 ± 5.3 ms, respectively; Table S1). The input resistance of VMN-*Wnt5a*-derived DA neurons *in vivo* (1039 ± 113 MΩ) was somewhat higher as compared to that both of VMN and VMN-*Wnt5a*-derived DA neurons in organotypic cultures. All recorded cells were retrospectively confirmed to be TH-GFP positive, by staining for biocytin infused during whole cell recordings (Fig. S3B).

Seven months after *in vitro* grafting onto slice cultures, VMN-*Wnt5a*-derived neurons were still present and viable in the slices. Their membrane properties were largely similar to those of VMN-*Wnt5a*-derived neurons in the cultures after 3-5 weeks (Table S1, Fig. S2 D-G). Average series resistance was 19.9 ± 1.5 MΩ, input resistance was 582.2 ± 91.6 MΩ and resting membrane potential was -59.9 ± 3.0 mV. Five of six recorded cells were spontaneously firing action potentials at a frequency of 2.3 ± 0.7 Hz (Fig S2D). Action potential amplitude was 82.2 ± 3.9 mV, rheobase 88.5 ± 3.9 pA, after-hyperpolarization amplitude and duration 13.9 ± 1.8 mV and 14.4 ms, respectively (Table S1). Sag was still not observed at this time point (Fig. S2G). Some changes as compared to 3-5 weeks time-point were slightly higher action potential threshold (-33.3 ± 1.4 mV; assessed by current step depolarizations) and half-width (0.8 ± 0.1 ms). Spontaneous EPSCs were present in all measured neurons, however, outward hyperpolarizing postsynaptic currents were not observed in any of these cells (Fig. S2E, F).
